# Over expression of toll-like receptors in peripheral blood and synovial fluid monocytes of enthesitis related arthritis category of juvenile idiopathic arthritis (JIA-ERA) patients contributes to secretion of inflammatory mediators

**DOI:** 10.1186/ar3651

**Published:** 2012-02-09

**Authors:** Arpita Myles, Amita Aggarwal

**Affiliations:** 1 Department of Clinical Immunology, Sanjay Gandhi Postgraduate Institute of Medical Sciences Lucknow-226014, India

## Background

TLRs 2, 4 and 9 have been implicated in murine models and human patients of arthritis, but the other TLRs are not well-investigated. Thus, we studied TLR expression and signaling and effect of TLR ligand stimulation in peripheral blood (PB) and synovial fluid (SF) monocytes (MC) of ERA patients.

## Methods

Levels of TLR2, TLR4 and TLR9 were measured by flow cytometry in ERA PBMC (n = 26), paired SFMC (n = 13) and healthy PBMC (n = 19) Real time PCR was done for TLRs 1-9 and their adaptors IRAK1, IRAK4, TRIF, TRAF3, TRAF6. PBMC and SFMC were stimulated with ligands for TLR1 (pam3-cys), 2 (peptidoglycan), 3 (polyI:C), 4 (LPS), 5 (flagellin) and 6 (zymosan). Levels of IL-6, IL-8 and MMP3 (ng/ml) were measured in the culture supernatants.

## Results

ERA PBMC had higher MFI of TLR2 [295.5(48.1-598) vs 179(68.7-442); p < 0.05] and TLR4 [448(178-2581) vs 402(229-569); p < 0.05] compared to controls. Intracellular TLR9 expression showed no significant difference between both groups. In paired samples, SFMC had higher MFI of both TLR2 [485(141-1683) vs 353(180-598); p < 0.05] and TLR4 [1016(42.4-3159) vs 513(193-2581); p < 0.05] compared to PBMC. Difference in TLR9 expression was not significant [1]. Patient PBMC (compared to healthy control) and SFMC (compared to corresponding PBMC) had higher RNA expression of TLRs1, 2, 3, 4, 5 and 6 and downstream adaptors. Patients PBMC produced significantly higher IL-6 (13.51 vs 6.54) and MMP3 (61 vs 32.9) as compared to controls on stimulation by LPS. With peptidoglycan also IL-6 (30.58 vs 10.84) and MMP-3 (102.54 vs 49.45) was higher than controls. Patient PBMCs produced more IL-6 and IL-8 compared to healthy PBMCs on stimulation with Pam3-cys, poly I:C, flagellin and zymosan. In paired samples, SFMCs showed a trend towards higher IL-6 and IL-8 production compared to PBMCs (Table 1)

**Table 1:** Production of IL-6 and IL-8 [median (range) ng/ml] by PBMCs and SFMCs upon TLR ligand stimulation.

Cultured with	IL-6			IL-8		
	
	Normal PBMC (n = 5)	ERA PBMC (n = 7)	ERA SFMC (n = 3)	Normal PBMC (n = 5)	ERA PBMC (n = 7)	ERA SFMC (n = 3)
Medium	4.4 (1.5-5.4)	7.6 (3-16.6) *	18 (9.3-24.2)	10 (4.3-12.6)	12.6 (8.1-35.7)	10 (4.3-12.6)
TNF	13.6 (9.6-14.8)	16 (12-35)	21(18-30)	30.8 (15.8-36.3)	34.4 (30.8-46.1)	31 (15.8-36.3)
Pam3cys	15.1 (13.3-19.6)	44 (26-62) **	53 (28-71)	37.1 (11.4-41.8)	106 (42.6-147.6) **	37.1 (11.4-42)
PolyI:C	13.1 (1.3-25.8)	28 (24-4) *	46 (45-65)	33.6 (31.1-56.3)	126(78-167) *	34(31.1-56.3)
Flagellin	14.1 (6.7-23.5)	34.9 (15-39.2) *	52.6 (40-53.8)**^#^**	35.2 (16.1-84.2)	115 (73-162) **	35 (16.1-84.2)
Zymosan	14.7 (8.8-36.3)	34 (28.4-39) *	48 (39.8-56.1)**^#^**	56 (12.6-89.6)	106 (103-163) **	56 (12.6-90)

p < 0.05** 0.01 ERA PBMC versus control PBMC p <^#^0.05 ERA SFMC versus ERA PBMC.

## Conclusion

Increased TLR expression and signaling on PBMC and SFMC from JIA-ERA patients may exacerbate disease by upregulating IL-6, IL-8 and MMP-3 in response to microbial/ endogenous ligands. TLR pathway is a potential therapeutic target in these patients.
